# Differential requirements of hippocampal *de novo* protein and mRNA synthesis in two long-term spatial memory tests: Spontaneous place recognition and delay-interposed radial maze performance in rats

**DOI:** 10.1371/journal.pone.0171629

**Published:** 2017-02-08

**Authors:** Takaaki Ozawa, Kazuo Yamada, Yukio Ichitani

**Affiliations:** Institute of Psychology and Behavioral Neuroscience, University of Tsukuba, Tsukuba, Ibaraki, Japan; Florida State University, UNITED STATES

## Abstract

Hippocampal *de novo* mRNA and protein synthesis has been suggested to be critical for long-term spatial memory. However, its requirement in each memory process (i.e. encoding, consolidation and retrieval) and the differences in the roles of *de novo* mRNA and protein synthesis in different situations where spatial memory is tested have not been thoroughly investigated. To address these questions, we examined the effects of hippocampal administration of the protein synthesis inhibitors, anisomycin (ANI) and emetine (EME), as well as that of an mRNA synthesis inhibitor, 5,6-dichlorobenzimidazole 1-β-D-ribofuranoside (DRB), on rat performance in two long-term spatial memory tests. In a spontaneous place recognition test with a 6 h delay, ANI, administered either before or immediately after the sample phase, but not before the test phase, eliminated the exploratory preference for the object in a novel place. This amnesic effect was replicated by both EME and DRB. In a 6 h delay-interposed radial maze task, however, administering ANI before the first-half and before the second-half, but not immediately or 2 h after the first-half, impaired performance in the second-half. This disruptive effect of ANI was successfully replicated by EME. However, DRB administered before the first-half performance did not impair the second-half performance, while it did impair it if injected before the second-half. None of these drugs caused amnesic effects during the short (5 min)/non-delayed conditions in either tests. These results suggest that 1) hippocampal protein synthesis is required for the consolidation of spatial memory, while mRNA synthesis is not necessarily required, and 2) hippocampal mRNA and protein synthesis requirement for spatial memory retrieval depends on the types of memory tested, probably because their demands are different.

## Introduction

The hippocampus is considered to play an essential role in spatial cognition and spatial memory. The requirement of hippocampal *de novo* mRNA and protein synthesis for the memory consolidation process, by which encoded short-term memory is transferred into long-term memory, is widely accepted. This has been repeatedly proven in many spatial tasks through the demonstration of the disruptive effects of mRNA synthesis inhibitors (mRNA-SIs) or protein synthesis inhibitors (PSIs) on performance in long, but not short, delay-interposed memory tasks [1–4]. However, the roles of *de novo* mRNA and protein synthesis in other memory processes, including retention and retrieval, are poorly understood. In previous studies, they have been examined only in the water maze task [5–7], the findings of which have led to the suggestion that hippocampal *de novo* protein synthesis is not associated with retrieval process.

Previous research on the neural basis of various processes involved in spatial memory suggests that the necessity for plasticity-related membrane receptor activity (e.g. α-amino-3-hydroxy-5-methyl-4-isoxazolepropionic acid (AMPA) and N-methyl-D-aspartate (NMDA) receptors) in each memory process can vary depending on the type of spatial memory task employed [8–11]. Considering these reports, the role of intra-hippocampal *de novo* mRNA and protein synthesis in each memory process might also be task-dependent. However, this has not been examined in previous studies.

To test this hypothesis, it is necessary to examine and compare the roles of *de novo* mRNA and protein synthesis in each memory process systematically, using multiple kinds of spatial memory tasks. In the present study, we chose to investigate this using two different kinds of spatial memory tests: the spontaneous place recognition (SPR) test and the delay-interposed radial arm maze (dRAM) task. These tasks are suitable for our study objective because they both consist of three different phases: acquisition, delay, and test phases. Therefore, each memory process can be separately investigated along the time axis [10]. However, it should be noted that these tests have different properties; SPR performance depends on incidental, non-associative learning/memory, and training or rule learning is not required, while dRAM performance largely depends on working memory, and training repetitions are required.

The present study investigated the roles of hippocampal *de novo* mRNA and protein synthesis in various processes of spatial memory. For this purpose, we adopted analogous task protocols for the two tests (SPR test and dRAM task), using the same delay length (6 h) and four time points for drug infusion (I: before acquisition phase, II: immediately (IIa) and 2 h (IIb) after acquisition phase, III: before test phase). This allowed for the comparison of the effects of intra-hippocampal administration of the broadly used PSIs, anisomycin (ANI) and emetine (EME), on performance. Additionally, we also tested the effect of an mRNA-SI, 5,6-dichlorobenzimidazole 1-β-D-ribofuranoside (DRB), at the time points at which ANI caused disruptive effects.

## Materials and methods

### Subjects

One hundred and eight male Wistar-Imamichi rats (Institute for Animal Reproduction, Ibaraki, Japan; 7–8 weeks) were used as subjects. Their mean body weight at the start of experiments was 274 g. Fifty-eight rats were assigned to the SPR test (experiment 1), and 50 rats were assigned to the dRAM task (experiment 2). In both experiments, animals were housed in individual cages on a 12:12 h light–dark cycle (light on: 0800–2000) with free access to water throughout the experiment. Feeding condition was different between the two experiments. Rats were kept with free access to food in experiment 1, while their feeding was limited to maintain 80–90% of their expected free feeding weight in experiment 2. Animal experiments were approved by the University of Tsukuba Committee on Animal Research (#09–025, #10–008). All efforts were made to minimize the number of animals used and their suffering.

### Surgery

Rats pretreated with atropine sulfate (0.05 mg, i.p.) were anesthetized with sodium pentobarbital (35 mg/kg, i.p.) and ketamine (10 mg, i.m.), and placed in a stereotaxic instrument (David Kopf Instruments, CA, USA). Guide cannulae were implanted bilaterally into the dorsal hippocampus with the stereotaxic coordinates (mm) AP: -3.8 from bregma, ML: ±2.7, DV: -3.0 from skull surface [12], and fixed on the skull with dental cement and small screws. Postoperative recovery period was 7 days.

### Drugs

Anisomycin (ANI; Wako, Osaka, Japan) was dissolved in ringer solution (Rin; Otsuka, Tokyo, Japan) by adding hydrochloride solution, and then the pH was adjusted to 7.2 by adding sodium hydroxide solution. Its final concentration was 50 or 100 μg/μl. 5,6-Dichlorobenzimidazole 1-β-D-ribofuranoside (DRB; Sigma, MO, USA) was diluted with 100% dimethyl sulfoxide (DMSO; Wako, Osaka, Japan) and then additionally diluted with saline (SAL, Otsuka, Tokyo, Japan). Its final concentration was 40 or 80 ng/μl in 2% DMSO in saline. Emetine (EME; Sigma, MO) was diluted with saline and brought to the final concentration, 32 or 64 μg/μl.

For intra-cerebral administration, drugs were bilaterally injected into the dorsal hippocampus via injection canulae, which were inserted into the guide cannulae and advanced 1.0 mm below the tips of them. The flow rate was kept 0.5 μl/min with a microsyringe pump (ESP-32, Eicom, Kyoto, Japan) for 2 min. After the drug injection, the injection cannulae were kept in place for additional 1 min to allow the diffusion of the drug. A previous study showed that 1 μl of ink into the cortex spread 1 mm on average [13]. Therefore, drugs are supposed to have diffused in whole hippocampal subregions including CA1 to CA3 and the dentate gyrus.

As for dosage of ANI, it has been shown that focal administration of 100 μg ANI into the gustatory cortex resulted in inhibition of the protein synthesis in more than 90% in the region, and lasted for more than 90 min [14]. Further, bilateral intracerebroventricular ANI of 100 μg suppressed protein synthesis in the hippocampus of more than 90% and it lasted at least for 45 min [2]. According to these studies, we infused 100 μg ANI as a higher dose. About DRB, in vitro, it has been shown that under the concentration of 100 μM (31.9 ng/μl) DRB solution, mRNA synthesis was suppressed by 75% [15]. Furthermore, in vivo, it has been reported that intra-hippocampal administration of 1.6 ng DRB decreased mRNA synthesis of brain-derived neurotrophic factor by 35% [16], and intra-amygdaloid administration of 10 ng DRB suppressed general mRNA synthesis by 53% [17]. Based on these previous studies, we decided to use 80 ng as a higher dose. The dose of EME was chosen according to previous studies showing that intracranial infusion of EME impaired LTM in rodents, in which dose of 8–50 μg were used [18, 19].

### Histology

After the behavioral tests, rats were deeply anesthetized with sodium pentobarbital (50 mg/kg, i.p.), and perfused intracardially with 0.02 M phosphate- buffered saline followed by 10% formalin solution (Wako, Osaka, Japan). The brains were further fixed in 10% formalin solution, then immersed in 20% sucrose solution. They were frozen by carbon dioxide, and sectioned in the coronal plane (40 μm) using a cryostat (CM3000, Leica, Heidelberg, Germany). Sections were Nissl-stained with cresyl violet (Chroma, Münster, Germany) to assess the location of the tips of injection cannulae.

### Spontaneous Place Recognition (SPR) test: Experiment 1

#### Apparatus

An open field arena (90 × 90 × 45 cm) made of polyvinyl chloride (O'Hara & Co., Ltd., Tokyo, Japan) was used, and its walls were colored in black, while the floor was gray. A white-black striped pattern was put on one of the sidewalls of arena as an absolute spatial cue. The objects employed were black and white triangular cast metals, black and white cylinders of cast metal, cans of juice, pink plastic cups and brown china bowls. All of them were heavy enough on their own or fixed on the heavy metal plate so that the rats could not move them. A video camera was suspended above the arena, and the image was projected to a screen allowing the experimenter to monitor and record the animals’ behavior.

#### SPR test procedure

**Fig 1:**
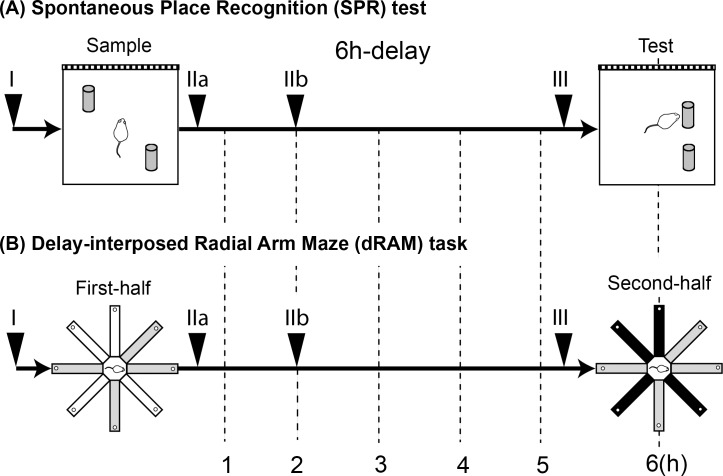
Schematic drawings of the spontaneous place recognition (SPR) test and the delay-interposed radial arm maze (dRAM) task. The delay length was 6 h in both tests. Roman numerals above each arrowhead show drug injection times. I: before the sample phase (or first-half performance); IIa: immediately after the sample phase (or first-half performance); IIb: 2 h after the sample phase (or first-half performance); III: before the test phase (or second-half performance). (A) In the SPR test, a white-black striped pattern was put on a sidewall of the arena as an absolute spatial cue. The test consisted of a sample phase (15 min), a delay period, and a test phase (5 min) and two identical objects were used throughout the test. In the test phase, one of the objects was moved to a novel position in the arena. (B) In the dRAM task, after the rat freely made four correct choices (gray arms), it was kept in a waiting cage. After the delay, it was required to visit the rest of arms (black arms) without reentering the arms visited already.

The SPR test was conducted a week after the surgery. Handling and habituation to the apparatus preceded the SPR test. The rats received 5-min handling for 4 days and 10-min habituation to the apparatus for 5 days. Schematic drawing of the procedure of the SPR test is shown in Fig 1A. One trial of the SPR test consisted of a sample phase (15 min) and a test phase (5 min). These phases were separated by a delay. In the present study, we used 5 min and 6 h delays. In the sample phase, two identical objects were placed in the diagonal corners of the arena (the center of each object was 22.5 cm from two adjacent walls), and the rat was allowed to explore the arena freely. The rat was then removed from the arena and taken to its home cage (delay period). After the delay, the rat was returned into the arena and allowed to explore freely (test phase). In this phase, one object was placed in the same position as in the sample phase (object *F*), while the other was moved to a different place (object *N*), which was 30 cm away from object *F* and 22.5 cm from a sidewall. After each phase, the floor of the arena and the objects were cleaned with 70% ethanol. After the experiment, an experimenter who was blind as to animal and treatment group assessed the time the rat spent exploring the objects. This was defined as the rat’s directing its nose at a distance within 2 cm from each object.

#### Drug tests in SPR test

In experiment 1–1, the effect of intra-hippocampal administration of ANI on a 6 h-delayed SPR test was examined first. Drugs were infused at four times within a trial: 30 min before the sample phase (time point I), immediately after the sample phase (time point IIa), 2 h after the sample phase (time point IIb), and 30 min before the test phase (time point III) (Fig 1A). Rats were assigned based on the time points of drug infusion (time point I, n = 10; time point IIa, n = 9; time point IIb, n = 10; time point III, n = 11). In the 6 h-delayed drug test, Rin (1 μl/side) and ANI (50 or 100 μg/1 μl/side) were tested in a random order. The effects of drugs were examined in a within-subject design. The interval between the drug tests was at least 48 h. After the 6 h-delayed test, the effect of ANI on the 5 min-delayed test was examined using ten randomly chosen rats. In the 5 min-delayed test, drugs were infused at 30 min before the sample phase, and the effects of Rin and a higher dose of ANI (100 μg/side) were tested in a random order. The test interval was the same as in the 6 h-delayed test.

In experiment 1–2, the effect of intra-hippocampal administration of DRB on the 6 h-delayed SPR test was examined (n = 10). Drugs were infused 60 min before the sample phase. In the 6 h-delayed drug test, 2% DMSO (1 μl/side) and DRB (40 or 80 ng/1 μl/side) were tested in a random order. After the 6 h-delayed test, the 5 min-delayed test was conducted using the same ten rats. In the 5 min-delayed test, the procedure of drug administration was the same as in the 6 h-delayed test, except that only 2% DMSO and a higher dose of DRB (80 ng/side) were tested.

In experiment 1–3, the effect of intra-hippocampal administration of EME on the 6 h-delayed SPR test was examined using animals tested in experiment 1–2 (n = 9, one rat was excluded because of damage to the guide cannulae). Drugs were infused 30 min before the sample phase. In the 6 h-delayed test, SAL (1 μl/side) and EME (32 or 64 μg/1 μl/side) were tested in a random order. After the 6 h-delayed test, the 5 min-delayed test was conducted using the same rats. In the 5 min-delayed test, the procedure of drug administration was the same as in the 6 h-delayed test, except that only the SAL and higher dose of EME were tested.

### Delay-interposed Radial Arm Maze (dRAM) task: Experiment 2

#### Apparatus

An elevated eight-arm radial maze made of black polyvinyl chloride (O'Hara & Co., Ltd., Tokyo, Japan) was used. The maze consisted of an octagonal center platform (32 cm in diameter) and 8 arms (60 cm × 12 cm) that radiated from the platform. A food well (1 cm in diameter, 0.5 cm deep) was carved out at each end of the arms, in which a food reward was baited (20 mg, TestDiet, IN, USA). Plexiglas guillotine doors (15 cm high) divided the arms from the center platform. The sidewalls of the arms were 4 cm high, except 12 cm from guillotine doors (12 cm high). A high transparent wall (24 cm×38 cm) was set on a side of the central end of each arm to prevent rats from climbing over the wall to directly move to the next arm without returning to the center platform. The maze was elevated 70 cm above the floor. There were extra-maze visual cues (e.g. sink, desk, calendar and door) around the maze in the experiment room. The illumination of central platform was 50 lux.

#### Radial maze task procedure

In this task, animals were trained for non-delayed task first, then further trained for delay-interposed task so that they ultimately could perform a 6 h delay-interposed task. Details of the training procedure were as follows.

(1) *Training of non-delayed task*. After 3 days of handling (5 min per day) and habituation to the maze (10 min per day), subjects were trained in a non-delayed radial maze task for one trial a day. One reward pellet was placed in each food well, and the subject was required to obtain all eight pellets without reentering the arms that had been previously visited in the trial. The subject was first placed in the center platform with guillotine doors closed. All eight guillotine doors were opened at once, and the subject was allowed to enter one of the arms and consume a pellet. When the rat returned to the center platform, all the doors were closed and it was confined for 5 s; then all eight guillotine doors were opened again to allow animals to make a next choice. This confinement was adopted to prevent rats from taking a specific strategy such as entering adjacent arms successively. One trial was finished when the subject took all eight pellets, made 16 choices, or 10 min had passed. Subjects had been trained until they reached the criterion of seven or eight correct choices in the first eight choices on five consecutive trials.

(2) *Training of delay-interposed task*. After training of the non-delayed task, rats were trained in the dRAM task. A schematic drawing of the procedure of the dRAM task is shown in Fig 1B. In this task, one trial was given a day, but subjects were taken out from the maze after the fourth correct choice and were put into the waiting cage. Animals never showed reentry errors (“within–half” errors) within the first half of trials in this study. After the delay period, they were returned back to the center platform and allowed to conduct the rest of the trial (second-half performance) to consume the other four pellets. The acquisition criterion was three or four correct choices in the first four choices of the second-half performance for five consecutive trials. First, subjects were trained for the 0 min-delayed task (they were put into the waiting cage and immediately returned back to the maze). After they acquired the 0 min-delayed task, they were trained for the 10 min-delayed task, and then further trained for the 6 h-delayed task. To reach acquisition criterion of the 6 h-delayed task, animals were trained for 53.4 ± 1.86 trials in total (i.e. non-delayed and delay-interposed tasks).

#### Drug tests in dRAM task

After the rats acquired the 6 h-delayed task, they received implantation of guide cannulae aiming for the dorsal hippocampus. Then rats were trained in the 6 h-delayed task again and after they reached the criterion (11.3 ± 1.07 trials), the drug test was conducted. Among each drug test (different doses of inhibitors vs. their vehicle) in each experiment (i.e. ANI, DRB and EME), all animals were retrained without drug infusion so that animals reach re-acquisition criteria of three or four correct choices in the first four choices of the second-half performance for two consecutive trials. In the present study, animals were retrained for 3.1 ± 0.28 trials among each drug test in each experiment.

In experiment 2–1, the effect of the intra-hippocampal administration of ANI on the performance in the 6 h-delayed task was examined. Drugs were infused at one of four time points within a trial: 30 min before the first-half (time point I), immediately after the first-half (time point IIa), 2 h after the first-half (time point IIb), and 30 min before the second-half (time point III) (Fig 1B). Rats were assigned based on the times of drug infusion (time point I, n = 10; time point IIa, n = 9; time point IIb, n = 8; time point III, n = 9). In the drug test on the 6 h-delayed task, Rin (1 μl/side) and ANI (50 or 100 μg/1 μl/side) were tested in a random order. The effects of drugs were examined in a within-subject design. Rats were trained at least in two drug-free trials between each drug test.

After the 6 h-delayed drug test, 21 rats were randomly chosen and assigned to either the state-dependency test (n = 12) or the drug test in the non-delayed task (n = 9). The state-dependency effect [20] of ANI was tested in the 6 h-delayed task. In the state-dependency test, drugs were infused at both time points of I and III. Before the drug test in the non-delayed task, the rats were trained on the non-delayed task again until they reached the criterion, and then the effect of ANI on the performance was tested. In the drug test in the non-delayed task, drugs were infused 30 min before the task. In these two tests, Rin and a higher dose of ANI (100 μg/side) were tested in a random order.

In experiment 2–2, the effect of intra-hippocampal administration of DRB on the performance in the 6 h-delayed task was examined. Drugs were infused at either of two time points within a trial, 60 min before the first-half (time point I, n = 12), or 60 min before the second-half (time point III, n = 10). 2% DMSO (1 μl/side) and DRB (40 or 80 ng/1 μl/side) were tested in a random order.

In experiment 2–3, the effect of intra-hippocampal administration of EME on the performance in the 6 h-delayed task was examined using animals tested in experiment 2–2 (n = 19, three rats were excluded because of damage to the guide cannulae). Drugs were infused at two times within a trial, 30 min before the first-half (time point I, n = 10), or 30 min before the second-half (time point III, n = 9). SAL (1 μl/side) and EME (32 or 64 μg/1 μl/side) were tested in a random order.

After the drug test at time points I and III, rats were randomly reassigned to either the drug test at time point IIa (n = 9) or the state-dependency test (n = 10). In the drug test at time point IIa, the procedure was the same as in the drug test at time points I and III except that drugs were infused immediately after the first-half performance. The state-dependency effect of EME was tested in the same procedure as in the state-dependency test of ANI in experiment 2–1.

Nine rats were randomly chosen after the drug test at time point IIa and the state-dependency test. The effect of intra-hippocampal administration of EME on the performance in the non-delayed task was tested. In contrast to experiment 2–1, rats were not retrained in the non-delayed task. In this drug test, drugs were infused 30 min prior to the trial. SAL and a higher dose of EME were tested in a random order. Rats were trained in at least two drug-free trials between each drug test.

### Statistical analysis

In the present study, all statistical analyses were performed using SPSS Statistics (IBM) and Graphpad Prism (GraphPad Software, Inc). Normality and equality of variances were assessed using Shapiro-Wilk test and Levene test, respectively. Normally distributed data sets were analyzed using parametric tests such as one-sample t-test, paired t-test and repeated measures ANOVA followed by Bonferroni post-hoc test. Whenever the assumption of normality or the equality of variances was violated, non-parametric tests, particularly Wilcoxon signed-rank test and Friedman test followed by a post-hoc Dunn’s multiple comparisons, were used. All data are presented as the average ± SEM.

## Results

### Histology

**Fig 2:**
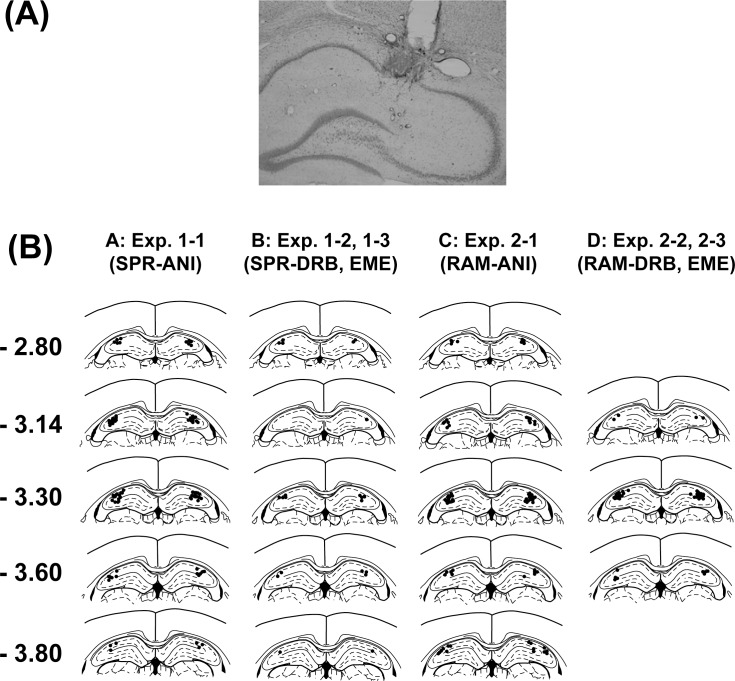
Locations of the tips of injection cannulae. Drugs were infused into the dorsal hippocampus in both experiments 1 and 2. (A) A photomicrograph showing a typical example of injection site. (B) Black dots denote tips of injection cannulae in each animal. Numbers in the left represent anteroposterior distance (mm) from bregma [12].

In the present study, all drugs were bilaterally injected into the dorsal hippocampus via the injection cannulae. The locations of tips of injection cannulae are shown in Fig 2B. All tips were identified in the dorsal hippocampus both in the SPR test (Fig 2B, A-B) and the radial maze task (Fig 2B, C-D) experiments.

### Experiment 1: Effects of hippocampal administration of protein and mRNA synthesis inhibitors on SPR performance

In the SPR test, for the assessment of the discrimination in the test phase, a discrimination ratio (DR) was calculated by dividing the amount of exploration of the object *N* in the test phase by the total amount of exploration for the two objects (object *F* + *N*). Only the exploration in the first minute was analyzed, because the novelty of object *N* was shown to decrease with time and the novelty preference could no longer be reliably assessed [21, 22].

#### Experiment 1–1: Effects of ANI

**Fig 3:**
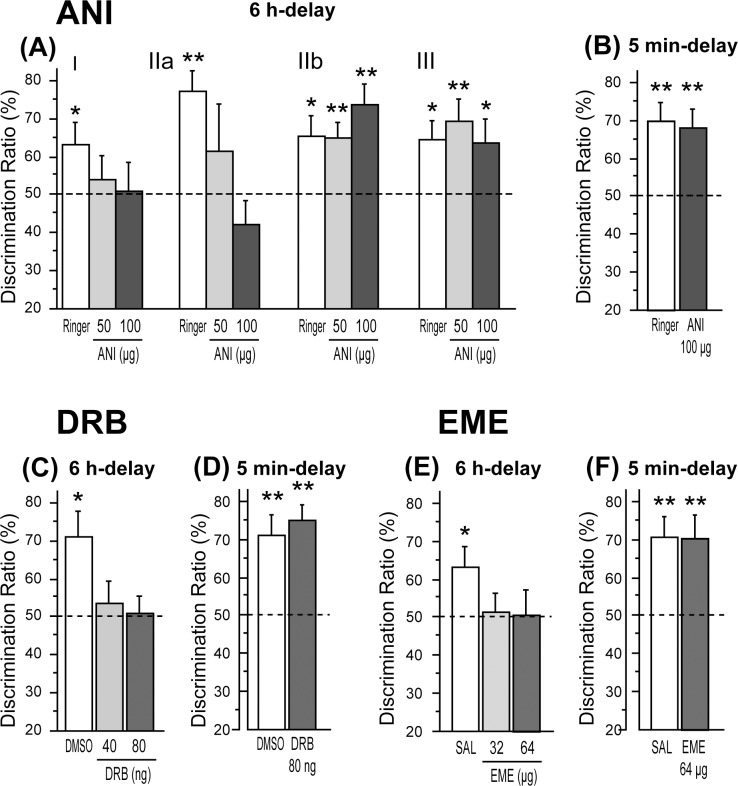
Effects of ANI, DRB and EME on the discrimination performance in the spontaneous place recognition test with 6 h and 5 min-delays. Effects of ANI (A, B), DRB (C, D), and EME (E, F) for the 6 h-delay (A, C, E) and the 5 min-delay (B, D, F). Data are shown as mean ± SEM. * p < .05, ** p < .01 vs. chance (50%).

To investigate the role of hippocampal protein synthesis, the effects of the intra-hippocampal administration of ANI were investigated. DRs in the test phase of the drug test of ANI with a 6 h delay are shown in Fig 3A. The treatment of ANI in the time points I and IIa diminished the test phase preference in a dose-dependent manner. According to a Friedman test at each time point, there was not any significant effect of ANI intra-hippocampal infusion at any time point. However, one-sample t-test revealed that DRs were above theoretical chance (50%) only under the control Rin condition in the time points I and IIa [I-Rin, t(9) = 2.53, p < .05; IIa-Rin, t(8) = 5.29, p < .01], while DRs under all drug conditions were above chance in the time points IIb and III [IIb-Rin, t(9) = 3.00, p < .05; IIb-ANI 50, t(9) = 3.89, p < .01; IIb-ANI 100, t(9) = 4.26, p < .01; III-Rin, t(10) = 2.95, p < .05; III-ANI 50, t(10) = 3.62, p < .01; III-ANI 100, t(10) = 2.43, p < .05].

DRs in the test phase of the drug test of ANI with the 5 min delay are shown in Fig 3B. In contrast to the results in the 6 h-delayed test, ANI did not have any effects on the preference in the 5 min-delayed test. As a result of a paired t-test, it was shown that DRs were not different between Rin and ANI. DRs were above chance in both conditions [Rin, t(9) = 4.07, p < .01; ANI 100, t(9) = 3.52, p < .01].

#### Experiment 1–2: Effects of DRB

To investigate the requirement of hippocampal gene transcription in memory processes in the SPR test, the effects of intra-hippocampal administration of DRB were examined. Since mRNA itself should not have a function in memory processes without translation, only DRB treatment before the sample phase, in which ANI showed a disruptive effect on the test phase performance, was examined in experiment 1–2. DRs in the test phase of the drug test of DRB with a 6 h delay are shown in Fig 3C. Treatment of DRB eliminated novel place preference in the test phase. According to a one-way ANOVA, there was not a significant effect of DRB. However, DR was above chance only under control DMSO treatment [t(9) = 3.08, p < .05], while DRs under both two doses of DRB treatments were not above chance. In the 5 min-delayed drug test (Fig 3D), a paired t-test revealed that DRs in the test phase were not different between DMSO and DRB. It was shown that DRs were above chance in both conditions [DMSO, t(9) = 4.09, p < .01; DRB 80, t(9) = 6.58, p < .01].

#### Experiment 1–3: Effects of EME

To confirm whether the disruptive effect of ANI on SPR performance can be replicated by another inhibitor of translation, the effects of intra-hippocampal administration of EME before the sample phase was tested. DRs in the test phase of the EME drug test with a 6 h delay are shown in Fig 3E. Treatment of EME eliminated the novel place preference in the test phase. According to a one-way ANOVA, there was not a significant effect of EME. However, DR was above chance only under the control SAL condition [t(8) = 2.36, p < .05], while DRs under both two doses of EME treatment conditions were not above chance. In the drug test with a 5 min delay (Fig 3F), a paired t-test revealed that DRs in the test phase were not different between SAL and EME. It was also shown that DRs were above chance in both conditions [SAL, t(8) = 3.71, p < .01; EME 64, t(8) = 3.47, p < .01].

### Experiment 2: Effects of hippocampal administration of protein and mRNA synthesis inhibitors on radial maze performance

In 8-arm radial maze task, rats were required to consume all 8 pellets placed at each end of arms without reentering the arms that had been previously visited in the same trial. In the dRAM task, one trial was separated by a 6 h delay into two parts, the first-half performance (the first four correct choices) and the second-half performance (the remaining four correct choices) (Fig 1B). Drugs were injected at the same four time points (I-III) as those of the 6 h-delayed SPR test. Only the second-half performance of a trial was analyzed, since rats did not show any errors in the first-half performance. Additionally, the effects of drugs infused before the beginning of trial on the performance in the non-delayed radial maze task were investigated in experiment 2–1 and 2–3.

#### Experiment 2–1: Effects of ANI

**Fig 4:**
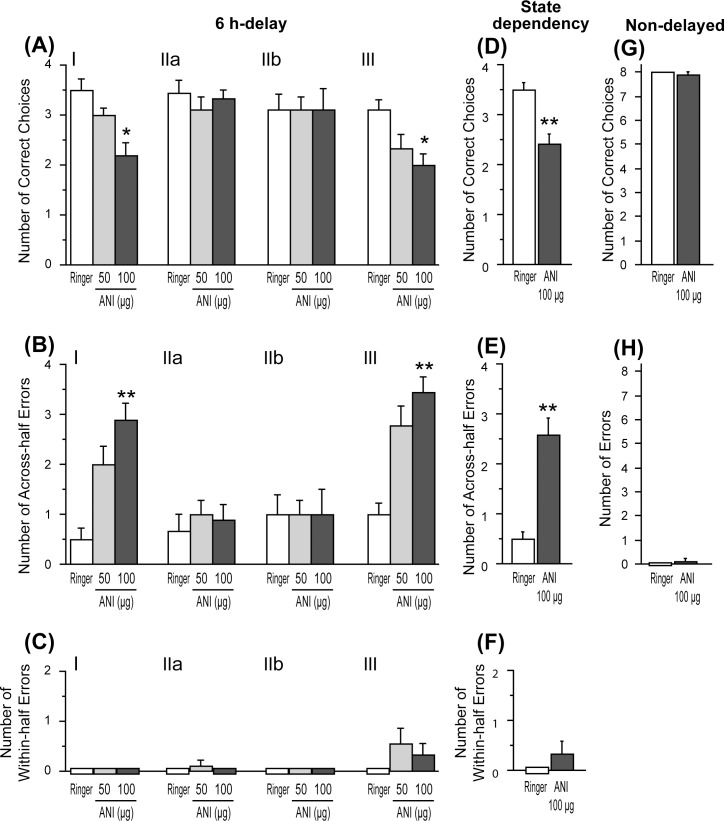
Effects of ANI on the second-half performance in the 6 h delay-interposed radial arm maze task. (A-C) Effects of ANI on the second-half performance in the 6 h-delayed task. (D-F) Effects of ANI on the second-half performance in the state-dependency test. (G-H) Effects of ANI on performance in the non-delayed radial arm maze task. (A, D) Number of correct choices in the first four choices. (B, E) Number of across-half errors. (C, F) Number of within-half errors. (G) Number of correct choices in the first eight choices. (H) Number of errors. Data are shown as mean ± SEM. * p < .05, ** p < .01 vs. Ringer condition.

The second-half performance in the drug test of ANI with a 6 h delay is shown in Fig 4A–4F). Treatment of ANI decreased the mean number of correct choices in the first four choices of the second-half in a dose-dependent manner when the drug was injected at time points I and III, but not at the time points IIa and IIb (Fig 4A). According to a Friedman test at each time point, there were significant effects of drug ANI injection at time points I [χ^2^(2) = 11.21, p < .05] and III [χ^2^(2) = 6.5, p < .05]. Subsequent post-hoc test revealed that at time points I and III the number of correct choices under the higher dose of ANI was fewer than that of Rin (p < .05).

Errors in the second-half performance were classified into two types and they were analyzed separately. Fig 4B shows the mean number of “across-half” errors, defined as reentering arms that had been visited in the first-half. Therefore, the maximum number of this type of error was four. Treatment of ANI increased the number of across-half errors in a dose-dependent manner when the drug was injected at the time points I and III, but not at the time points IIa and IIb. A Friedman test at each time point revealed significant effects of ANI when it was injected at time points I [χ^2^(2) = 15.94, p < .0001] and III [χ^2^(2) = 13.15, p < .01], and subsequent post-hoc test revealed that the number of errors under the higher dose of ANI was higher than that of Rin (p < .01) in time points I and III.

Fig 4C shows the mean number of “within-half” errors defined as reentering arms that had already been visited within the second-half performance. A small number of within-half errors was seen only in time points IIa and III, but not in time points I and IIb. Friedman test revealed that there was not a difference in the number of errors in time points IIa and III.

In experiment 2–1, intra-hippocampal administration of ANI before the first-half or second-half performance impaired the second-half performance, suggesting a possibility that the disruptive effect of ANI could be attributed to the ‘state dependency effect’ [20]. To investigate this possibility, we examined whether intra-hippocampal administration of ANI at both times I and III impairs the second-half performance or not. A Wilcoxon signed-rank test revealed that ANI significantly decreased the number of correct choices in the first four choices (Fig 4D) [W = 45, p < .01], and increased the number of “across-half” errors (Fig 4E) [W = -66, p < .01] but did not have effect on the number of “within-half” errors in the second-half performance (Fig 4F).

Fig 4G and 4H show the effects of ANI on the non-delayed task performance. A Wilcoxon signed-rank test revealed that there was no difference of the number of the correct choices and errors between Rin and ANI conditions.

#### Experiment 2–2: Effects of DRB

**Fig 5:**
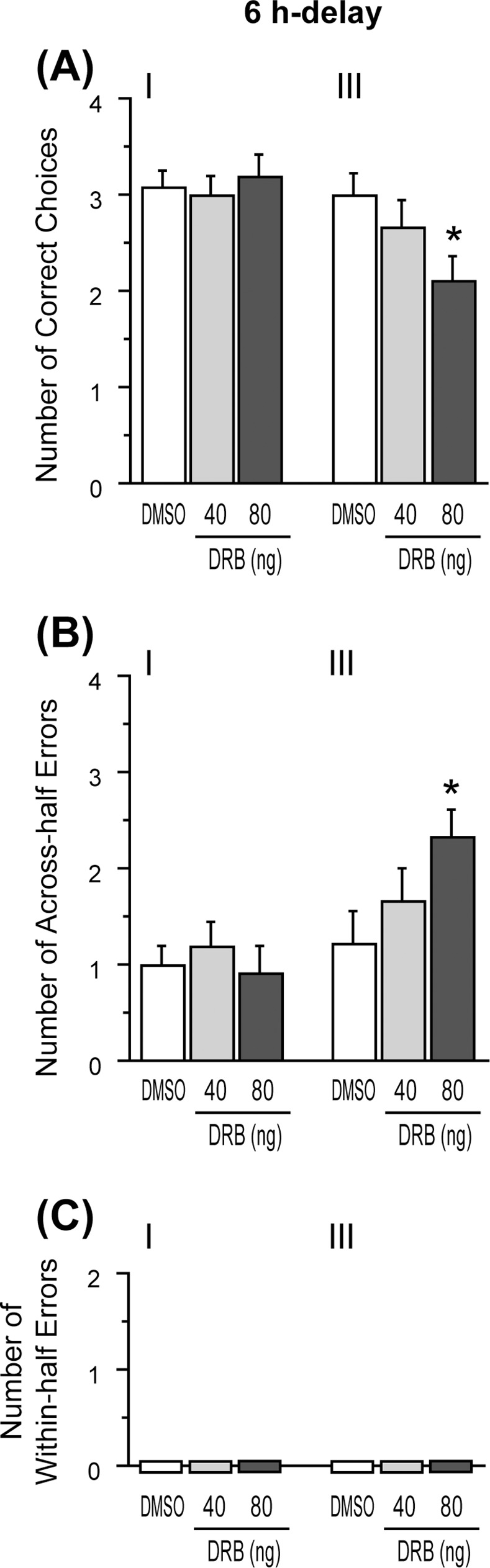
Effects of DRB on the second-half performance in the 6 h delay-interposed radial arm maze task. (A) Number of correct choices in the first four choices. (B) Number of across-half errors. (C) Number of within-half errors. Data are shown as mean ± SEM. * p < .05 vs. DMSO condition.

The requirement of hippocampal gene transcription in memory processes in the radial maze task was examined using DRB. The second-half performance in the drug test of DRB with a 6 h delay is shown in Fig 5A–5C. A Friedman test at each time point revealed that DRB decreased the number of correct choices in the first four choices [χ^2^(2) = 10.38, p < .01], and increased “across-half” errors [χ^2^(2) = 9, p < .01] when injected at time point III, but not at time point I (Fig 5A and 5B). Subsequent post-hoc test revealed that under a higher dose of DRB, the number of correct choices was fewer and “across-half” errors were greater than that of DMSO (p < .05) at time point III. In this experiment, no rats made “within-half” errors under all drug conditions (Fig 5C).

#### Experiment 2–3: Effects of EME

**Fig 6:**
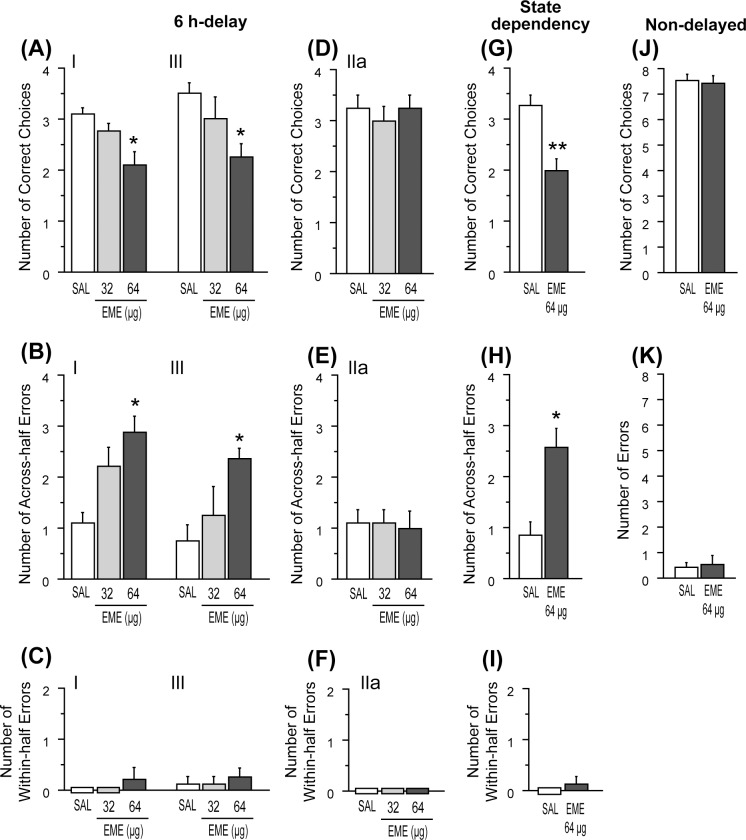
Effect of EME on the second-half performance in the 6 h delay-interposed radial arm maze task. (A-F) Effect of EME on the second-half performance in the 6 h-delayed task. (G-I) Effect of EME on the second-half performance in the state-dependency test. (J-K) Effect of EME on the performance in the non-delayed task. (A, D, G) Number of correct choices in the first four choices. (B, E, H) Number of across-half errors. (C, F, I) Number of within-half errors. (J) Number of correct choices in the first eight choices. (K) Number of errors. Data are shown as mean ± SEM. * p < .05, ** p < .01 vs. saline (SAL) condition.

In order to confirm whether disruptive effect of ANI on the radial maze performance could be replicated by another inhibitor of translation, the effects of intra-hippocampal administration of EME were tested. The number of correct choices in the first four choices (Fig 6A) and the number of “across-half” errors in the second-half of the drug test of EME at time points I and III (Fig 6B and 6C) were analyzed using a Friedman test. It was revealed that at both time points, EME significantly changed both scores of correct choices [time point I, χ^2^(2) = 9.92, p < .01; time point III, χ^2^(2) = 8.67, p < .01] and “across-half” errors [time point I, χ^2^(2) = 12.62, p < .01; time point III, χ^2^(2) = 9.47, p < .01]. As a result of a subsequent post-hoc test, it was revealed that the number of correct choices under the higher dose of EME was less than that of SAL (p < .05), and the number of “across-half” errors under the higher dose of EME was greater than that of SAL (p < .05) at both time points. However, there was no difference of the number of “within-half” errors among drug conditions in each injection time. The number of correct choices in the first four choices (Fig 6D) and the number of “across-half” and “within-half” errors in the second-half of the drug test in time point IIa (Fig 6E) were also analyzed by a Friedman test, and it was revealed that EME did not change any scores (Fig 6F).

Similar to ANI, EME before the first-half or second-half impaired the second-half performance, suggesting the possibility of a state dependency effect of EME. To investigate this possibility, we tested the effect of intra-hippocampal administration of EME at both times I and III on the second-half performance. According to a Wilcoxon signed-rank test under the higher dose of EME, the number of correct choices was lower [W = 55, p < .01] and the number of “across-half” errors was higher [W = -35, p < .05] than that of SAL (Fig 6G and 6H). On the other hand, a Wilcoxon sighed rank test revealed that there was no difference in the number of “within-half” errors between SAL and EME (Fig 6I).

Fig 6J and 6K show the effects of EME on the non-delayed task performance. A Wilcoxon signed-rank test revealed that there were no differences in the number of correct choices in the first eight choices and the number of errors between SAL and EME.

## Discussion

**Fig 7:**
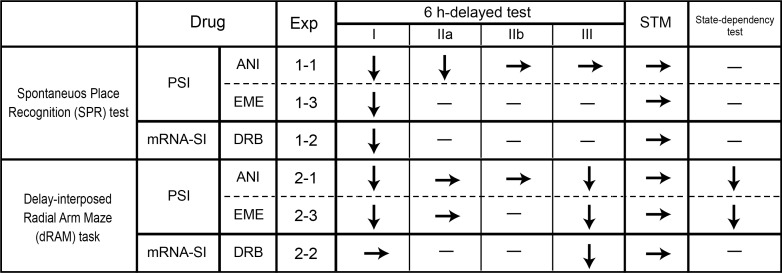
Summary of the results of the present study. ↓: impairment by the inhibitor. →: no impairment.–: not examined. PSI: protein synthesis inhibitor. mRNA-SI: mRNA synthesis inhibitor. Short-term memory (STM) in the SPR test was estimated through the effect of drugs on performance with a 5 min-delay. In the radial maze task, STM was estimated using the effect of drugs on performance in the non-delayed task or on the number of within-half errors in the 6 h-delayed task.

In the present study, it was found that the effects of hippocampal administration of PSIs (ANI and EME) and an mRNA-SI (DRB) on memory test performance were largely different between the SPR test and the dRAM task, depending on the time of treatment. The results are summarized in Fig 7.

### The role of hippocampal *de novo* mRNA and protein synthesis in the encoding and consolidation processes

#### *De novo* protein synthesis in the encoding and consolidation processes

In the SPR test, intra-hippocampal administration of PSIs before or immediately after the sample phase abolished the preference for the object in a novel place. The observed impairment by ANI administered immediately after the sample phase (time point IIa, Fig 3A) suggests a requirement of hippocampal protein synthesis in the consolidation process in the SPR test. However, the disruptive effect of the pre-sample phase injection of PSIs (time point I, Fig 3A and 3E) can be interpreted as an impairment of memory encoding and/or consolidation. Nevertheless, no disruptive effects were found with a delay period of 5 min (Fig 3B and 3F), suggesting that encoding and short-term maintenance of spatial memory in this task do not require hippocampal protein synthesis. Therefore, given this result, it can be considered that the effects of PSIs administered at the pre-sample phase were caused by an impairment of memory consolidation rather than one of encoding processes. It has been previously suggested that the consolidation of spatial memory, in various tasks assessing this process, requires hippocampal protein synthesis [1–3]. The present study has demonstrated this requirement in the SPR test for the first time.

Similarly, in the dRAM task, PSIs injected before the first-half impaired performance in the 6 h-delayed, but not in the non-delayed task (time point I, Fig 4A, 4B, 4G and 4H), suggesting the requirement of hippocampal protein synthesis in the consolidation process. However, we also found that the administration of ANI immediately after the first-half did not cause a disruptive effect (time point IIa, Fig 4A and 4B), unlike what was observed in the SPR test. In a previous study, intra-hippocampal (CA3 region) administration of ANI impaired performance in a 4 h-delayed, 12 arm dRAM task, if ANI was injected before, but not after, the first-half [1]. The pattern of disruptive effects of ANI observed in the present study is consistent with this previous result. A possible interpretation for this lack of disruptive effects induced by ANI, injected immediately after the first-half in dRAM task, is that protein synthesis-dependent memory consolidation might have already been completed by the time the injected ANI became effective. Bohbot et al. [23] suggested that spatial memory is consolidated very rapidly (within less than 15 seconds) in a delayed matching-to-place task in a water maze, which is thought of as a spatial working memory task. In accordance with this previous result, our findings seem to suggest that spatial memory is more rapidly consolidated in the dRAM task than that in the SPR test. However, results from both tests show that the process of consolidation, itself, requires protein synthesis.

Administration of ANI 2 h after the sample phase or first-half did not show any effects (time point IIb, Figs 3A, 4A and 4B) on the SPR or dRAM test performance, suggesting that, by this time, memory consolidation had been completed and that hippocampal protein synthesis is, thus, not required for memory retention. However, it has been reported that there are multiple time windows of sensitivity to PSIs in the interval between inhibitory avoidance training and its retention test [16], or during the retention interval of social recognition memory test [24, 25]. Thus, further investigation on the effects of PSIs, at various time points within delay periods, will need to be conducted in the future.

#### *De novo* mRNA synthesis in the encoding and consolidation processes

Similar to ANI, intra-hippocampal administration of DRB, before the sample phase, abolished the preference for the object in a novel place in the 6 h-delayed SPR test, but not in the 5 min-delayed test (Fig 3C and 3D). This suggests that mRNA synthesis is required for the consolidation process involved in the SPR test. This is consistent with a previous study that showed a requirement of hippocampal mRNA synthesis in the consolidation of spatial memory in a water maze task [4].

However, unlike the effect of ANI, hippocampal administration of DRB before the first-half (time point I) did not show any effects on the performance in the dRAM task (Fig 5A and 5B). This indicates that there is a possibility that spatial memory, in the dRAM task, is consolidated through a hippocampal protein synthesis-dependent, but mRNA synthesis-independent mechanism. In addition, the absence of disruptive effects induced by DRB does not seem to be attributed to the insufficiency of the selected dosage, because even the lowest dose used in the present study (40 ng/side) was much higher than that used in a previous study, where DRB successfully inhibited mRNA synthesis and disrupted memory consolidation in a fear conditioning task [17]. This discrepancy between the effects of PSIs and mRNA-SI detected in the current study is consistent with previous studies in which PSIs impaired retention, reconsolidation or extinction of aversive memory, while mRNA-SIs did not [17, 26–28]. However, this has never been reported for the initial phase of spatial memory consolidation. One possible explanation for the mRNA-independent memory consolidation mechanism in the dRAM task is that neural plasticity underlying consolidation might occur through the translation of pre-existing mRNA, before the beginning of the first-half performance. Importantly, several studies have suggested that mRNA can be dendritically localized and then translated at specific synapses when it becomes necessary [29], and that the translation of pre-existing mRNA is enough to establish long-term synaptic changes, without the need for new mRNA synthesis [30, 31].

### The role of hippocampal *de novo* mRNA and protein synthesis in the retrieval process

In the SPR test, the injection of ANI before the test phase did not impair the subsequent discrimination performance at all (time point III in Fig 3A) suggesting that in SPR performance, the retrieval of spatial memory does not require newly synthesized protein in the hippocampus. Supporting this result, previous studies using the water maze task suggested that hippocampal protein synthesis is not important for memory retrieval [5–7]. Conversely, in the dRAM task, both ANI and DRB injected into the hippocampus at time point III significantly impaired performance in the second-half (Figs 4A, 4B, 5A and 5B). This suggests that the retrieval of long-term spatial memory is a process that requires *de novo* mRNA and protein, which are synthesized at the time point when second-half choice behavior, based on the information from the responses generated 6 h earlier, is expressed. The treatment of these inhibitors increased the number of across-half errors, but not that of within-half errors, in the second-half performance (Figs 4C and 5C). This suggests that spatial cognition or short-term spatial memory was not disrupted by the protein/mRNA synthesis inhibitor and that rats were normally motivated for the task. As for the requirement of protein synthesis for memory retrieval, the involvement of *de novo* protein synthesis in the memory retrieval process has rarely been reported. However, Lopez et al. [32] have suggested that ongoing protein synthesis is required for AMPA receptor trafficking, which takes place during retrieval of fear memories in the amygdala. Therefore, the present study may have found, for the first time, that there are situations where hippocampal *de novo* protein synthesis is necessary for the retrieval of long-term, but not short-term, spatial memory.

Our present results raise a question about the differential requirements of *de novo* protein or mRNA synthesis for memory retrieval between the two tests that our study employed. Indeed, the effects of pre-test injections of a PSI in the SPR test were the same as those found in the water maze task in previous studies [5–7], where rats did not need working memory to perform. Given that the mechanisms of consolidation/retrieval have been studied, mainly using working memory-independent tasks, one possible interpretation of the present results could be that the working memory component of the dRAM task caused non-canonical requirements of *de novo* protein or mRNA synthesis for memory retrieval.

Another important difference between SPR and dRAM learning is that in the dRAM task, rats were repeatedly trained before any drugs were administered. Therefore, it is plausible that well-trained rats knew when the information that had been acquired in the first-half performance would be needed to solve the task. Thus, the predictability of the second-half of the task, for the animals performing it, was unique to the dRAM task, and this fact seems to have made new protein synthesis necessary for memory retrieval. In contrast, in the SPR test, there was no way for the rats to be able to predict the timing of test phase in which they showed preferred exploration of the object in a novel place. However, in order to test this hypothesis, we need further studies in which there is stricter control of the predictability of the time point at which the animals’ second-half choice response will be tested after a delay.

### The effects of protein synthesis inhibitors

In the dRAM task, performance in the second-half was impaired when ANI and EME were administered either at time point I or III, while their administration at time points IIa and IIb did not show any effects. These results could be interpreted as a ‘state dependency’ effect that comes from the change of drug states between the first- and second-halves of a trial [20]. However, these PSIs impaired the second-half performance even if they were injected both at time points I and III, in which the drug states between the first and second halves of a trial were the same (Fig 4D and 4E, Fig 6). Therefore, the effects of ANI could not be attributed to the ‘state dependency’ effect.

As for the disruptive effect of ANI on the consolidation and/or retrieval process, it should be mentioned that besides protein synthesis inhibition, ANI also has a stimulatory effect on neurotransmitter release, which can induce time-dependent memory disruption [33, 34]. Therefore, we also examined the effect of EME [18, 19, 35–37], another broadly used PSI. This drug completely replicated the amnesic effects observed using ANI in both tests, supporting the possibility that the disruptive effects of ANI and EME are due to their primary effect on protein synthesis. However, further investigation, including the knockdown of specific genes involved in neuronal plasticity or memory will be needed to clarify the differential requirement of hippocampal mRNA/protein synthesis during the SPR test and the dRAM task.

## Conclusions

The present study investigated the roles of hippocampal *de novo* mRNA and protein synthesis in various processes of spatial memory, using the SPR test and the dRAM task. Results suggest that 1) hippocampal protein synthesis is required for the consolidation of spatial memory, while mRNA synthesis is not necessarily required, and that 2) the requirement of hippocampal mRNA and protein synthesis for spatial memory retrieval depends on the types of memory tested, probably because they each have different demands.

## Abbreviations

ANI: anisomycin
EME: emetine
DRB: 5,6-dichlorobenzimidazole 1-β-D-ribofuranoside
DMSO: dimethyl sulfoxide
SAL: saline
SPR: spontaneous place recognition
dRAM: delay-interposed radial arm maze
PSI: protein synthesis inhibitor
mRNA-SI: mRNA synthesis inhibitor
